# Defining the relationship between pain intensity and disease activity in patients with rheumatoid arthritis: a secondary analysis of six studies

**DOI:** 10.1186/s13075-022-02903-w

**Published:** 2022-09-10

**Authors:** Fowzia Ibrahim, Margaret Ma, David L. Scott, Ian C. Scott

**Affiliations:** 1 Centre for Rheumatic Diseases, Department of Inflammation Biology, School of Immunology and Microbial Sciences, Faculty of Life Sciences and Medicine, King’s College London, Cutcombe Road, London, SE5 9RJ UK; 2 Level 10, Tower Block, Division of Rheumatology, University Medicine Cluster, National University Health System, 1E Kent Ridge Road, Singapore, 119228 Singapore; 3 Department of Medicine, Yong Loo Lin School of Medicine, National University of Singapore, Singapore, 119228 Singapore; 4 Primary Care Centre Versus Arthritis, School of Medicine, Keele University, Keele, UK; 5 Haywood Academic Rheumatology Centre, Haywood Hospital, Midlands Partnership NHS Foundation Trust, High Lane, Burslem, Staffordshire UK

**Keywords:** Rheumatoid arthritis, Pain intensity, Disease activity assessment, Remission

## Abstract

**Background:**

Pain is the main concern of patients with rheumatoid arthritis (RA) while reducing disease activity dominates specialist management. Disease activity assessments like the disease activity score for 28 joints with the erythrocyte sedimentation rate (DAS28-ESR) omit pain creating an apparent paradox between patients’ concerns and specialists’ treatment goals. We evaluated the relationship of pain intensity and disease activity in RA with three aims: defining associations between pain intensity and disease activity and its components, evaluating discordance between pain intensity and disease activity, and assessing temporal changes in pain intensity and disease activity.

**Methods:**

We undertook secondary analyses of five trials and one observational study of RA patients followed for 12 months. The patients had early and established active disease or sustained low disease activity or remission. Pain was measured using 100-mm visual analogue scales. Individual patient data was pooled across all studies and by types of patients (early active, established active and established remission). Associations of pain intensity and disease activity were evaluated by correlations (Spearman’s), linear regression methods and Bland-Altman plots. Discordance was assessed by Kappa statistics (for patients grouped into high and low pain intensity and disease activity). Temporal changes were assessed 6 monthly in different patient groups.

**Results:**

A total of 1132 patients were studied: 490 had early active RA, 469 had established active RA and 173 were in remission/low disease activity. Our analyses showed, firstly, that pain intensity is associated with disease activity in general, and particularly with patient global assessments, across all patient groups. Patient global assessments were a reasonable proxy for pain intensity. Secondly, there was some discordance between pain intensity and disease activity across all disease activity levels, reflecting similar discrepancies in patient global assessments. Thirdly, there were strong temporal relationships between changes in disease activity and pain intensity. When mean disease activity fell, mean pain intensity scores also fell; when mean disease activity increased, there were comparable increases in pain intensity.

**Conclusions:**

These findings show pain intensity is an integral part of disease activity, though it is not measured directly in DAS28-ESR. Reducing disease activity is crucial for reducing pain intensity in RA.

## Introduction

Pain remains a major challenge for patients with rheumatoid arthritis (RA). Most patients experience regular pain of at least moderate intensity [1, 2]. Although patients emphasise the importance of controlling their pain [3], reducing disease activity is the dominant goal of current specialist management [4]. Measuring disease activity provides a single score calculated by combining diverse clinical assessments. The main disease activity scores combine tender and swollen joint counts, acute phase marker measurements like the erythrocyte sedimentation rate (ESR) and patient global assessments (PtGA). They omit assessments of pain intensity, which creates an apparent paradox. It means RA patients’ main concern, pain, appears to be ignored by specialists’ focus on reducing disease activity.

International pain management guidelines in inflammatory arthritis highlight the need to reduce disease activity to minimise RA pain, aiming for remission or low disease activity (LDA) [5]. Despite such recommendations, several key evidence gaps exist in understanding how pain intensity is related to disease activity. Our goal is to provide evidence to address these gaps.

The first evidence gap is understanding the extent to which the different components of disease activity—swollen joint counts (SJC), tender joint counts (TJC), ESR levels and PtGA scores—drive its association with pain intensity. There is some evidence PtGA scores are strongly associated with pain intensity [6–8] while SJC and ESR are only weakly associated [9]. However, these relationships require better characterisation.

A second evidence gap is the extent of discordance between pain intensity and disease activity. Mean pain intensity scores are lower in groups of patients with RA in remission and LDA [10–12], but in real-world settings many individual patients in remission have substantial pain [13]. The extent of “discordance” between pain intensities and disease activity in moderate (MDA) and high disease activity (HDA) is unknown, though the existence of “rheumatoid robustus” patients with active disease but few symptoms suggest it may be fairly common [14].

A third evidence gap is whether a dynamic relationship exists between pain intensity and disease activity in which increases in pain intensities are accompanied by increases in disease activity and vice versa. Studies of their relationship are almost entirely cross-sectional analyses at single time-points [10–12]. Both measures are dynamic outcomes with marked within-individual variability over time [15, 16]. To be certain controlling disease activity reduces pain intensity requires establishing a dynamic temporal relationship between them.

We have addressed these three evidence gaps in secondary analyses of five trials and a longitudinal observational cohort. These studies spanned active early and established RA and patients with persisting remission and LDA. Our three aims comprised: (1) defining associations between pain intensity and disease activity components, (2) evaluating discordance between pain intensity and disease activity and (3) assessing temporal changes in pain intensity and disease activity.

## Methods

### Studies and patients

We identified randomised controlled trials and prospective observational studies which met the following criteria: we had designed the studies, patients were entered from 2000 onwards, the studies were grant-funded, they enrolled patients with RA, they lasted at least 12 months and baseline, six-month and 12-month data were collected on pain intensity scores, disease activity scores for 28 joints (DAS28) and disability. Six studies met these criteria; five were trials and one was an observational study. Two trials (CARDERA-1 [17] and CARDERA-2 [18]) enrolled patients with early active RA. Two trials (TACIT [19] and TITRATE [20]) enrolled patients with established active RA. One trial (OPTTIRA [21]) and one observational study (REMIRA [22]) enrolled patients in remission or LDA.

### Clinical assessments

Patients were assessed independently by specialist nurses or equivalent health care professionals who did not know in the trials what treatment they were receiving. In all studies, disease activity was assessed using the Disease Activity Score with the Erythrocyte Sedimentation Rate for 28 joints (DAS28-ESR) based on measurements of its four components. Pain intensity was measured using a 100-mm visual analogue scale (VAS). Additional baseline data evaluated included age, sex, disease duration and body mass index (BMI).

### Association between disease activity and pain intensity

We analysed this association at 12 months when the spread of disease activity was greatest by: (a) pooling all patients in the six studies and (b) pooling studies according to the types of patients enrolled as early active RA (CARDERA-1/CARDERA-2), established active RA (TACIT/TITRATE) and remission/LDA (OPTTIRA/REMIRA). These groups were selected as pain may be driven by different factors in early active RA (e.g. inflammation) compared to established RA (e.g. joint damage) and remission/LDA (e.g. non-inflammatory processes); grouping patients in this manner allowed us to examine relationships between disease activity and pain intensity in settings where pain pathogenesis may vary.

### Statistical analyses

The analytic approaches were as follows. First, mean pain intensity scores were reported in patients stratified by conventional disease activity categories (remission [DAS28-ESR <2.6]; LDA [DAS28-ESR 2.6 to <3.2]; MDA [DAS28-ESR ≥3.2 to ≤5.1]; HDA [DAS28-ESR >5.1]. Second, Spearman’s correlations between pain intensity scores and DAS28-ESR and its components were calculated (correlation coefficients of 0 to 0.19, 0.2 to 0.39, 0.40 to 0.59, 0.6 to 0.79, and 0.8 to 1 were considered very weak, weak, moderate, strong and very strong correlations, respectively [23]). Third, the degree of variance in pain intensity scores explained by DAS28-ESR and its components was evaluated using multivariable linear regression models. These included pain intensity scores at 12 months as the response variable, and DAS28-ESR at 12 months, age, gender, ethnicity, treatment (DMARDs vs. Biologics) and baseline scores for DAS28-ESR and pain intensity as explanatory variables. Regression models were repeated for each DAS28-ESR component (in place of DAS28-ESR). Finally, as PtGA and pain intensity VAS are on the same scale (0 to 100 units), we constructed Bland-Altman plots to examine the agreement between the two measures at the group- and individual-level [24]. The standard deviation (SD) of the difference between the pain intensity VAS and PtGA was used to estimate the limits of agreement (with 95% of the differences lying between these).

The relationship between pain intensity scores and disease activity was also assessed over a very wide range of disease activity states by dividing DAS28-ESR scores for all patients combined into 13 categories, which were in steps of 0.50 from ≤1.5 to >7.5. Mean pain intensity scores (95% CI) and pain categories (low ≤34, moderate 35–74 and high >74) were calculated for each of the 13 DAS28-ESR categories.

### Discordance between disease activity and pain intensity

DAS28-ESR and pain intensity scores were categorised into low, moderate or high levels, and the kappa statistic calculated. We considered kappa values of ≤0, 0.01 to 0.20, 0.21 to 0.40, 0.41 to 0.60, 0.61 to 0.80, and 0.81 to 1.00 as indicating none, none to slight, fair, moderate, substantial and almost perfect agreement, respectively [25]. For “low” disease activity levels, we pooled remission and LDA into a single group. For pain intensity, we used VAS cut-offs shown in patients with chronic musculoskeletal pain to best identify those describing their pain as “mild” (≤34), “moderate” (>34 to < 74) and “severe” (≥74) [26]). These analyses were undertaken at 12 months, and in all RA patients (pooling studies) and studies grouped by the types of patients enrolled (early active, established active, remission/LDA).

In addition, data from all patients was divided into three disease activity categories (LDA/remission, MDA and HDA) and three pain intensity score categories (low ≤34, moderate 35–74 and high >74). In each of these nine categories, mean (95% CI) levels of DAS28-ESR components (PtGA, tender and swollen joint counts, and ESR) were calculated.

### Impact of changing disease activity states on pain intensity scores over time

To examine the impact of moving between active (MDA/HDA) and inactive (LDA/remission) disease activity states over time, we allocated patients to one of four groups based on their DAS28-ESR scores at 6 and 12 months. The groups comprised DAS28-ESR levels of (1) >3.2 at 6 and 12 months (persistently active); (2) >3.2 at 6 months but ≤3.2 at 12 months (active then inactive); (3) ≤3.2 at 6 months but >3.2 at 12 months (inactive then active); (4) ≤3.2 at 6 and 12 months (persistently inactive). We reported mean pain intensity scores with 95% CIs at each time-point in studies stratified by the types of patients enrolled (early active trials, established active trials and remission/LDA studies).

We used three packages for different analyses: R (version 4.1.0), IBM SPSS (version 27) and STATA (version 16). All the analyses used original individual patient data which was combined across trials and studies to evaluate the different questions being addressed.

## Results

### Patients studied

A total of 1132 patients were studied (Table 1): 490 in early active RA trials (CARDERA-1/CARDERA-2), 469 in established active RA trials (TITRATE/TACIT) and 173 in remission/LDA studies (OPTTIRA/REMIRA). Most (66–82%) were female; mean ages were 54–57 years. Patients in early RA trials had short mean disease durations (0.1–0.3 years); those in established active RA trials and remission/LDA studies had longer mean disease durations (4–11 years).

**Table 1:** Patient characteristics and outcomes

	Early active RA trials		Established active RA trials		RA remission/LDA studies	
	*CARDERA-1*	*CARDERA-2*	*TITRATE*	*TACIT*	*OPTTIRA*	*REMIRA*
	*n = 355*	*n = 135*	*n = 292*	*n = 177*	*n = 85*	*n = 88*
Study type	Trial of methotrexate, ciclosporin and steroids	Trial of methotrexate and anakinra	Trial of biologic DMARDs and synthetic DMARDs	Trial of treat to target and routine care	Trial of biologic DMARD tapering	Observational study
**Demographics**						
Females, *n* (%)	241 (68%)	92 (68%)	238 (82%)	137 (77%)	63 (74%)	58 (66%)
Age (years)	54 (12)	55 (12)	57 (12)	57 (12)	57 (11)	57 (15)
Disease duration (years)	0.3 (0.4)	0.1 (0.2)	6 (6)	8 (9)	11 (12)	4 (3)
**Baseline outcome measures**						
28 TJC	11.2 (7.5)	16.9 (7.5)	7.5 (4.1)	16.9 (6.7)	0.4 (0.8)	0.6 (1.0)
28 SJC	9.6 (6.2)	11.7 (6.9)	4.1 (2.7)	10.6 (6.5)	0.4 (1.0)	1.2 (2.0)
ESR	42 (30)	39 (23)	16 (14)	32 (25)	13 (13)	11 (9)
PtGA	55 (27)	58 (28)	46 (20)	68 (20)	10 (11)	25 (21)
DAS28-ESR	5.8 (1.3)	6.4 (1.2)	4.4 (0.5)	6.3 (0.8)	1.9 (0.8)	2.2 (0.9)
Pain intensity VAS	46 (25)	55 (28)	42 (22)	66 (20)	11 (14)	23 (25)
**12-month outcome measures**						
28 TJC	5.2 (5.6)	8.6 (9.1)	5.6 (5.5)	6.4 (7.6)	2.1 (3.7)	0.8 (1.6)
28 SJC	7.1 (7.8)	4.1 (5.8)	2.7 (3.5)	3.3 (4.4)	1.3 (2.5)	1.0 (2.2)
ESR	28 (22)	23 (20)	16 (16)	23 (23)	14 (13)	12 (11)
PtGA	36 (27)	33 (28)	35 (26)	40 (30)	18 (20)	27 (24)
DAS28-ESR	4.3 (1.6)	4.2 (1.8)	3.6 (1.4)	3.9 (1.6)	2.5 (1.3)	2.2 (1.1)
Pain intensity VAS	32 (26)	36 (30)	33 (28)	40 (29)	20 (21)	26 (26)

Data presented as means (standard deviations) unless otherwise stated; *n*, number; *TJC*, tender joint count; *SJC*, swollen joint count; *ESR*, erythrocyte sedimentation rate; *PtGA*, patient global assessment; *VAS*, visual analogue scale; *DAS28-ESR*, disease activity score for 28 joints with the erythrocyte sedimentation rate

Baseline mean DAS28-ESR scores were 5.9 (95% CI 5.8, 6.0), 5.1 (95% CI 5.0, 5.2) and 2.0 (95% CI 1.9, 2.1) in the early and established active RA trials and remission/LDA studies. At 12 months, they were 4.3 (95% CI 4.1, 4.4), 3.7 (95% CI 3.6, 3.9) and 2.4 (95% CI 2.2, 2.5). Baseline mean pain intensity scores were 49 (95% CI 47, 51), 51 (95% CI 50, 52), and 17 (95% CI 14, 20) in the early and established active RA trials and remission/LDA studies. At 12 months, they were 33 (95% CI 31, 36), 35 (95% CI 33, 38) and 22 (95% CI 19, 26).

### Association between disease activity and pain intensity

#### Mean pain intensity scores by disease activity states

**Fig. 1:**
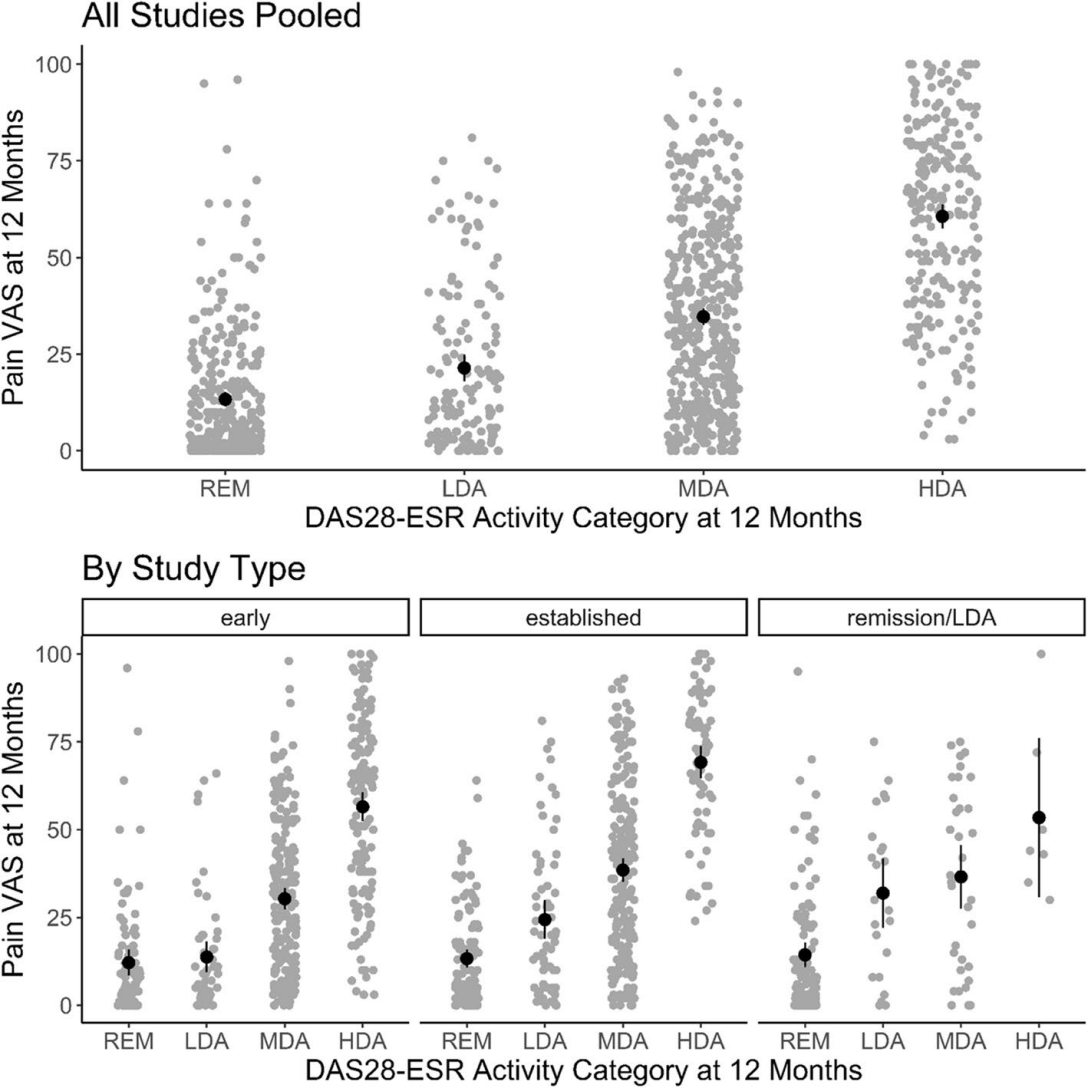
Mean pain intensity scores stratified by disease activity categories at 12 months. Mean scores are represented by the black dots, and 95% confidence intervals by vertical black bars. Each patient’s pain VAS score is plotted as grey points, with “jitter” applied across the horizontal axis to prevent overplotting; early = early active RA trials; established = established active RA trials; remission/LDA = remission/LDA studies; REM = remission; LDA = low disease activity; MDA = moderate disease activity; HDA = high disease activity

Mean pain intensity scores rose as disease activity levels increased (Fig. 1). When all studies were pooled, 12-month mean pain intensity scores were 13 (95% CI 12, 15), 21 (95% CI 18, 25), 35 (95% CI 33, 37) and 61 (95% CI 58, 64) in patients in remission, LDA, MDA and HDA. However, superimposed plots of each patient’s pain VAS score (Fig. 1) showed there was a substantial spread of 12-month pain intensity scores in each category of disease activity. The pain scores formed a low cluster in remission/LDA; in contrast, they formed a high cluster in HDA. There was a broader spread of pain scores across the whole range in MDA. The same patterns were seen when patients were grouped by study type. Mean pain intensity scores were lowest in remission (range 12–14) and highest in HDA (range 53–69).

#### Spearman’s correlations

**Fig. 2:**
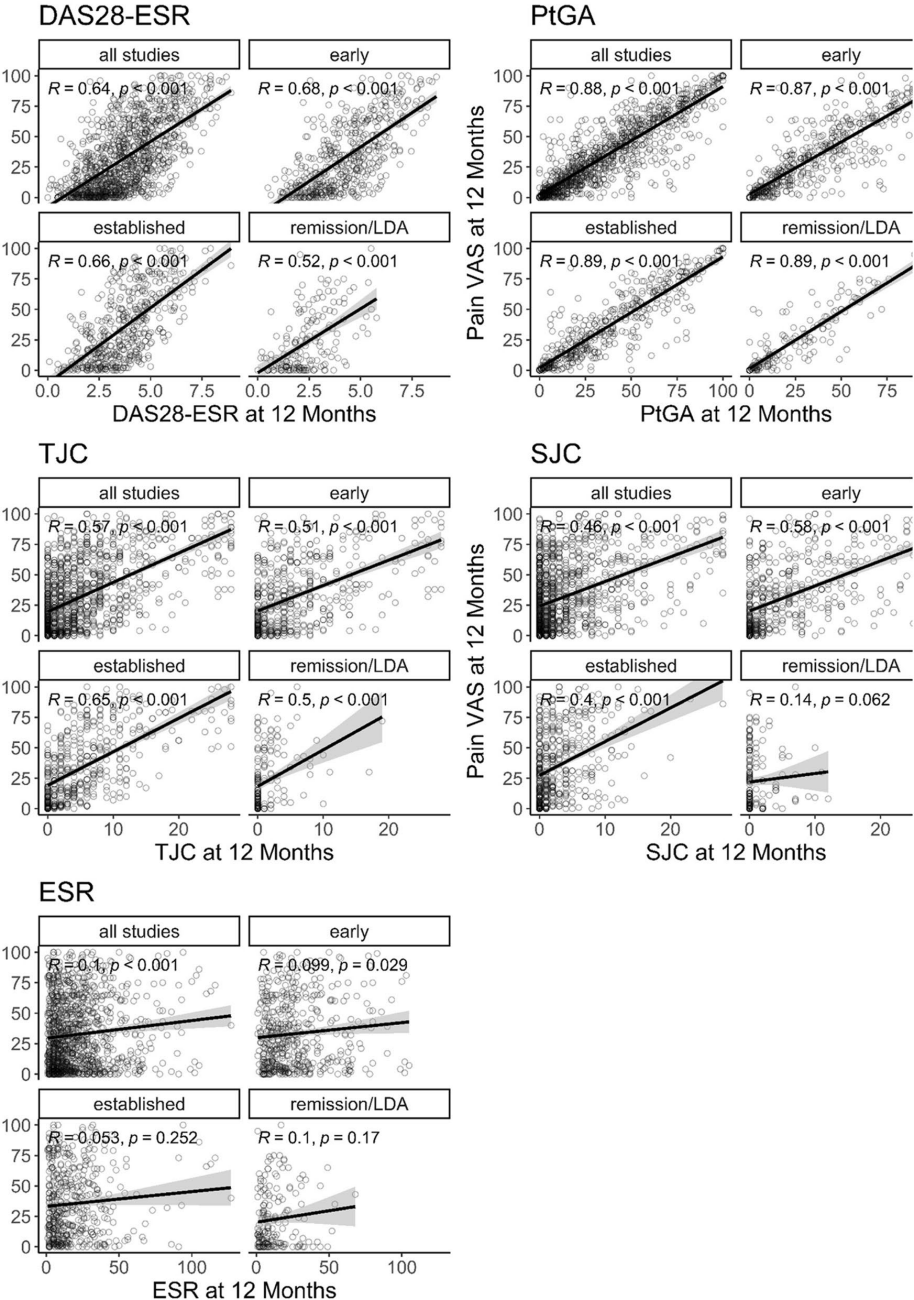
Scatterplots demonstrating relationship between pain intensity scores and DAS28-ESR and its components at 12 months. All studies = all studies pooled; early = early active RA trials; established = established active RA trials; remission/LDA= remission/LDA studies. Spearman’s rank correlation coefficients with *P*-values are given for the variables plotted on each scatterplot and linear regression lines plotted

There were strong correlations between 12-month pain intensity and DAS28-ESR scores (Fig. 2) in all studies pooled (*r* = 0.64). When patients were grouped by study type correlations were stronger in early (*r* = 0.68) and established (*r* = 0.66) active RA trials than in remission/LDA studies (*r* = 0.52). The strength of correlations between pain intensity VAS and DAS28-ESR components varied considerably. They were strong with PtGA (*r* = 0.87 to 0.89), moderate to strong with TJC (*r* = 0.50 to 0.65), very weak to moderate with SJC (*r* = 0.14 to 0.58) and very weak with ESR (*r* = 0.05 to 0.10).

#### Linear regression models

Analysis of data at 12 months using linear regression models showed DAS28-ESR and its components all had statistically significant associations with pain intensity scores in all studies pooled (Table 2). In established active RA trials, the SJC was not significantly associated with pain intensity scores; in remission/LDA studies, the SJC and ESR were not significantly associated with pain intensity scores. In all settings, in adjusted models, the PtGA explained most of the variance in pain intensity scores (76% to 86%). In contrast, ESR levels explained just 21% of the variance in pain intensity scores in early and established active RA trials.

**Table 2:** Linear regression models examining relationships between pain intensity scores and DAS28-ESR and its components at 12 months

	Unadjusted			Adjusted		
	*β (95%CI)*	*p-value*	*R*^*2*^	*β (95%CI)*	*p-value*	*R*^*2*^
***All studies (n=1,132)***						
DAS28-ESR	10.71 (10.0, 11.4)	<0.001	0.423	11.5 (10.7, 12.2)	<0.001	0.534
Patient global	0.89 (0.86, 0.92)	<0.001	0.774	0.86 (0.83, 0.89)	<0.001	0.793
Tender joint counts	2.41 (2.20, 2.61)	<0.001	0.319	2.20 (1.98, 2.42)	<0.001	0.410
Swollen joint counts	2.02 (1.78, 2.27)	<0.001	0.188	1.99 (1.73, 2.25)	<0.001	0.325
ESR	0.15 (0.06, 0.23)	<0.001	0.011	0.20 (0.11, 0.29)	<0.001	0.197
***Early active RA trials (n = 490)***						
DAS28-ESR	11.26 (10.21, 12.31)	<0.001	0.477	10.61 (9.52, 11.71)	<0.001	0.539
Patient global	0.86 (0.81, 0.90)	<0.001	0.737	0.82 (0.77, 0.87)	<0.001	0.759
Tender joint counts	2.08 (1.78, 2.38)	<0.001	0.277	1.72 (1.42, 2.03)	<0.001	0.382
Swollen joint counts	2.05 (1.78, 2.32)	<0.001	0.313	1.88 (1.61, 2.16)	<0.001	0.412
ESR	0.12 (0.01, 0.24)	0.033	0.009	0.14 (0.03, 0.25)	0.016	0.205
***Established active RA trials (n=469)***						
DAS28-ESR	12.41 (11.13, 13.68)	<0.001	0.439	12.62 (11.35, 13.89)	<0.001	0.507
Patient global	0.91 (0.87, 0.96)	<0.001	0.795	0.89 (0.84, 0.93)	<0.001	0.801
Tender joint counts	2.75 (2.44, 3.06)	<0.001	0.391	2.65 (2.33, 2.98)	<0.001	0.426
Swollen joint counts	2.77 (2.16, 3.39)	<0.001	0.143	2.64 (1.99, 3.28)	<0.001	0.214
ESR	0.12 (−0.01, 0.26)	0.077	0.007	-	-	-
***Remission/LDA studies (n=173)***						
DAS28-ESR	10.51 (8.02, 12.99)	<0.001	0.289	10.02 (6.93, 13.11)	<0.001	0.481
Patient global	0.94 (0.87, 1.01)	<0.001	0.792	0.91 (0.82, 1.00)	<0.001	0.859
Tender joint counts	3.00 (1.84, 4.16)	<0.001	0.132	2.40 (1.28, 3.52)	<0.001	0.381
Swollen joint counts	0.70 (−0.83, 2.24)	0.368	0.005	-	-	-
ESR	0.19 (−0.10, 0.48)	0.201	0.010	-	-	-

*DAS28-ESR*, disease activity score for 28 joints with the erythrocyte sedimentation rate; adjusted model contains the covariates age, gender, disease duration, body mass index, baseline pain VAS intensity score, baseline score for DAS28-ESR or the individual components baseline score, and treatment

#### Agreement between pain intensity VAS and PtGA

**Fig. 3:**
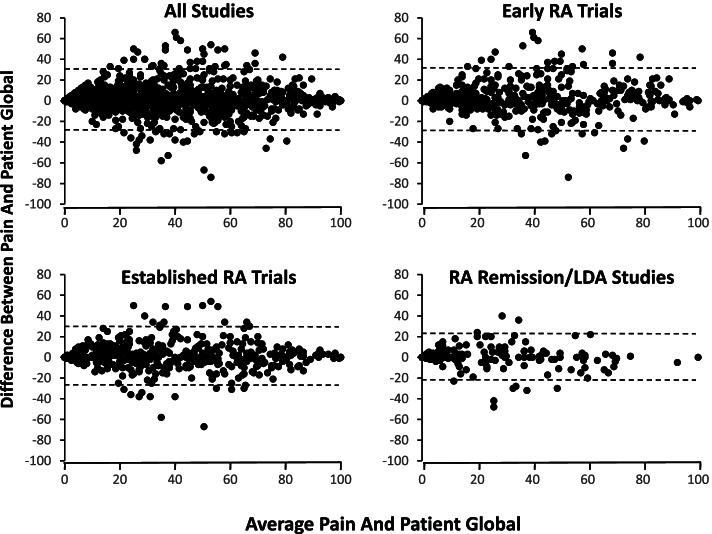
Bland-Altman plots of 12-month pain intensity scores and patient global assessments. Pain = pain intensity visual analogue scale scores; dashed lines show upper and lower limits of agreement

Bland-Altman plots showed pain intensity VAS scores were very similar to PtGA scores with mean pain scores only 1.6 units higher than mean PtGA scores in all studies pooled (Fig. 3). This finding highlights the extent to which these two measures provide similar findings. Mean differences were larger in early active RA trials (mean difference 2.3) and established active RA trials (mean difference 1.4) than remission/LDA studies (mean difference 0.1). Differences between pain intensity VAS and PtGA were least at the high and low ends of measurement. There were substantial differences between scores in individual patients whose mean of these scores approached 50 units.

#### Relationships across broad range of disease activity levels

The relationship between pain intensity and DAS28-ESR remained when DAS28-ESR scores were subdivided into 13 categories (≤1.50 to >7.50). Mean pain intensity scores increased incrementally from 8 (95% CI 6, 10) with DAS28-ESR ≤1.5, to 83 (95% CI 78, 89) with DAS28-ESR >7.5. For each 0.5 increase in DAS28-ESR, pain intensity scores increased by a mean of 6, though the actual increase across all DAS28-ESR levels ranged from 1 to 11.

### Discordance between disease activity and pain intensity

#### Agreement between pain intensity and DAS28-ESR

The agreement between disease activity groups and low, moderate or high pain intensity scores was only “fair”. Kappa statistics for all studies pooled and by study type ranged from 0.263 to 0.307 (Table 3). Although most patients in remission/LDA had low pain intensity levels (81–92% across study types), a considerable number (7–17% across study types) had moderate pain levels. In addition, many patients in MDA also had low pain intensity levels (45–59% across study types), and many patients in HDA had only moderate pain intensity levels (44–71% across study types). Similar patterns of agreement were seen in all study types.

**Table 3:** Agreement between disease activity and pain categories at 12 months

Pain intensity VAS score categories	DAS28-ESR categories			Kappa statistic
	*Remission/low (≤3.2)*	*Moderate (>3.2 to 5.1)*	*High (>5.1)*	
***All studies***				
*Low (≤34)*	388 (86%)	241 (54%)	41 (18%)	
*Moderate (35–74)*	59 (13%)	170 (38%)	111 (48%)	0.287
*High (>74)*	6 (1%)	37 (8%)	79 (34%)	
***Early active RA trials***				
*Low (≤34)*	130 (92%)	118 (59%)	32 (22%)	0.263
*Moderate (35–74)*	10 (7%)	75 (38%)	72 (49%)	
*High (>74)*	2 (1%)	6 (3%)	43 (29%)	
***Established active RA trials***				
*Low (≤34)*	151 (84%)	105 (49%)	8 (10%)	0.307
*Moderate (35–74)*	26 (15%)	79 (37%)	34 (44%)	
*High (>74)*	2 (1%)	29 (14%)	35 (46%)	
***Remission/LDA studies***				
*Low (≤34)*	107 (81%)	15 (46%)	1 (14%)	0.295
*Moderate (35–74)*	23 (17%)	16 (49%)	5 (71%)	
*High (>74)*	2 (2%)	2 (6%)	1 (14%)	

*DAS28-ESR*, disease activity score for 28 joints with the erythrocyte sedimentation rate; *VAS*, visual analogue scale; all kappa statistic values have *P*-values <0.001

The relationship between pain categories and disease activity continued to show evidence of some discordance when DAS28-ESR scores were subdivided into 13 categories. Only patients with DAS28-ESR scores ≤2.0 had no high pain scores; 165/178 (93%) had low pain scores and 13/178 (7%) had moderate pain scores. Only patients with DAS28-ESR scores >7.0 had no low pain scores; 14/39 (36%) had moderate pain scores and 25/39 (64%) had high pain scores. All 13 DAS28-ESR categories included some patients with moderate pain scores.

#### Pain categories and different DAS28-ESR components

The discordance between pain intensity and disease activity in some individuals appeared to reflect varying contributions of the different components of DAS28-ESR scores to overall disease activity (Table 4). In patients in LDA or remission, the minority of patients with high pain scores also had relatively high PtGA scores and TJCs; in contrast, the majority of patients in LDA or remission with low pain scores also had low PtGA scores and TJCs. Similarly, the minority of patients with HDA with low pain scores also had relatively low PtGA scores and TJCs; in contrast, the majority of patients with moderate or high pain scores also had high PtGA scores and TJCs.

**Table 4:** Mean DAS28-ESR component scores across pain categories at 12 months

DAS28-ESR category	Pain category	Number of patients	Patient global	Tender joints	Swollen joints	ESR
	*Low (≤34)*	388	12 (10, 13)	0.9 (0.7, 1.0)	0.7 (0.6, 0.9)	13 (12, 15)
*Remission/low (≤3.2)*	*Moderate (35–74)*	59	42 (37, 46)	2.0 (1.4, 2.5)	0.6 (0.3, 0.9)	7 (5, 9)
	*High (>74)*	6	66 (48, 84)	2.5 (0, 5.6)	0.2 (0, 0.6)	7 (0.1, 14)
	*Low (≤34)*	241	23 (21, 25)	5.1 (4.6, 5.6)	3.5 (3.0, 4.0)	26 (24, 28)
*Moderate (>3.2 to 5.1)*	*Moderate (35–74)*	170	49 (46, 51)	5.6 (4.9, 6.2)	3.9 (3.3, 4.6)	18 (17, 20)
	*High (>74)*	37	71 (65, 77)	8.3 (6.4, 10.3)	3.7 (2.7, 4.8)	10 (7, 12)
	*Low (≤34)*	41	37 (31, 43)	8.6 (6.9, 10.2)	10.9 (9.2, 12.7)	41 (33, 49)
*High (>5.1)*	*Moderate (35–74)*	111	58 (55, 61)	12.5 (11.1, 13.9)	11.0 (9.6, 12.4)	39 (34, 44)
	*High (>74)*	79	85 (83, 88)	16.7 (14.9, 18.5)	12.6 (10.7, 15.5)	33 (27. 38)

Means (95% confidence intervals) are shown; *DAS28-ESR*, disease activity score for 28 joints with the erythrocyte sedimentation rate

### Impact of changing disease activity states on pain intensity scores over time

**Fig. 4:**
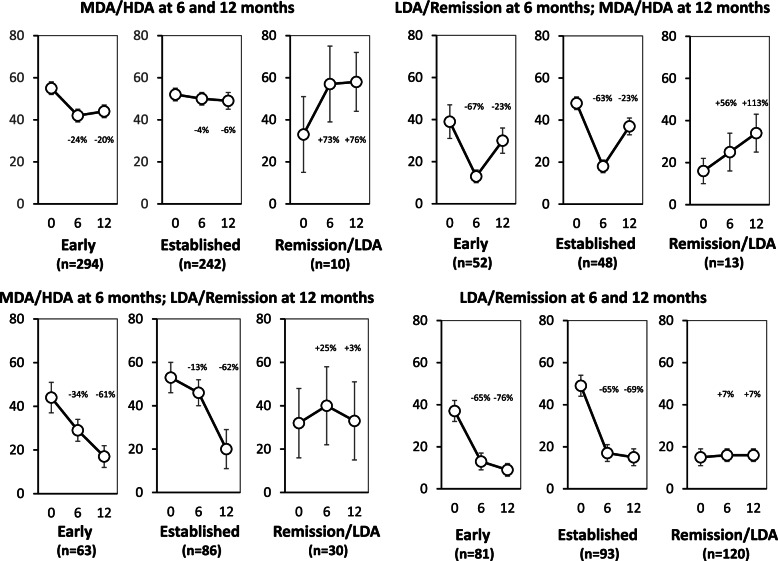
Mean pain intensity scores at 6 and 12 months in patients by disease activity status at each time-point. Error bars show 95% confidence intervals; percentage change in pain intensity scores from baseline is shown

Changes in pain intensity scores mirrored changes in disease activity scores at 6 and 12 months (Fig. 4). When patients had MDA or HDA at 6 or 12 months, they also had relatively high pain scores (means ranging from 29 to 58). When patients had LDA or remission at 6 or 12 months, they also had relatively low pain scores (means ranging from 9 to 33). Changing from MDA/HDA to LDA/remission resulted in a change from high to low pain scores and vice versa. In trials of early and established active RA, achieving LDA/remission at 6 or 12 months resulted in falls in mean pain scores from baseline of over 60%, but achieving MDA/HDA resulted in falls in mean pain scores of less than 35%. In remission/LDA studies, persisting LDA and remission resulted in increases in pain scores of less than 10%, but achieving MDA or HDA at 12 months resulted in increases in pain scores of more than 60%.

Patients in trials of early and established active RA all initially had MDA/HDA. Those patients in whom MDA/HDA persisted at 6 and 12 months also had persistently high pain scores (mean 55 and 52 initially, and 44 and 49 at 12 months). In contrast, patients in remission/LDA studies all had initial LDA/remission. Those patients with persisting LDA/remission at 6 and 12 months had persistently low pain scores (mean 15 initially, and 16 at 12 months). In contrast, patients in trials of early and established active RA who achieved LDA/remission at 6 and 12 months had low pain scores at both time points (mean 37 and 49 initially, 13 and 17 at 6 months, and 9 and 15 at 12 months). Patients in remission/LDA studies with MDA/HDA at 6 and 12 months had high pain scores at both time points (mean 33 initially, 57 at 6 months and 58 at 12 months). Patients in trials of early and established active RA who achieved LDA/remission at 6 months but then reverted to MDA/HDA at 12 months showed a fall then a rise in pain scores (mean 39 and 48 initially, 13 and 18 at 6 months, and 30 and 37 at 12 months).

## Discussion

Our secondary analysis of 1132 patients with RA followed over 12 months in five trials and one observational study shows three key findings. Firstly, pain intensity measured using a VAS is closely related to disease activity measured using DAS28-ESR. Pain levels mirrored disease activity levels in both cross-sectional and longitudinal analyses. Secondly, most patients in remission and LDA have low pain intensity scores but a considerable number of patients in MDA/HDA also report only low pain levels. Thirdly, the association between disease activity and pain intensity is largely attributable to the association between PtGA and pain VAS. Taken together, these findings strongly support targeting remission and LDA to minimise pain intensity with the caveat that not all patients with active RA experience substantial pain. The findings also suggest that pain itself is an integral part of the concept of “disease activity” with assessments of PtGA and pain intensity VAS measuring the same construct in many patients.

To our knowledge, our study is the first to examine the discordance between pain intensity and higher levels of disease activity; prior research has focused on pain scores in remission [13]. We found 46 to 59% of patients in MDA had low pain intensity levels and 44 to 71% of patients in HDA had moderate pain intensity levels. These findings reflect two concepts. First, pain in RA is driven by many factors in addition to inflammation and synovitis [27]. Second, the DAS28-ESR is a composite score with different components driving disease activity in different patients. Regardless of this finding, the evidence that achieving remission or LDA improves pain intensity as well as other outcomes like function, quality of life and radiological damage [12, 28, 29] is substantial and the case for treat-to-target remains compelling.

Our finding that PtGA and pain VAS are highly correlated and significantly associated replicates existing research in this field [6, 7] with Studenic et al. reporting that pain intensity scores explained 76% of the variance in PtGA scores [7]. Our Bland-Altman plots showing that agreement between PtGA and pain VAS is high when both scores are either high or low, but substantially less when the mean scores of PtGA and pain VAS are ~50 also replicates, existing research by Egmose et al. in the Danish DANBIO registry [8]. They reported that amongst 221 patients with RA, mean differences between PtGA and pain VAS were minimal at 5.2 units, but substantial for patients in whom the mean difference was ~50 (with upper and lower limits of agreement of 29.5 and −19.1). Taken together, our findings suggest that patients with PtGA-pain VAS concordance focus on pain intensity when answering PtGA, and patients with PtGA-pain VAS discordance focus on other aspects of their lives. Such variation in how people interpret the PtGA has been described in a mixed-methods study of 33 patients with RA, which reported that patients considered their pain, fatigue, function and psychological well-being, with comorbidities and RA sequelae also influencing scoring [30]. Other studies using quantitative approaches also demonstrate strong associations between PtGA and function, emotional distress and social participation [7, 8, 31]. In addition, the relationship between pain intensity and PtGA scores reflects previous research on the subjective components of disease activity scores by McWilliams and colleagues [32].

Unlike many other trials of synthetic, biologic and targeted synthetic DMARDs, the trials included in our secondary analysis not only recorded pain intensity, but also measured it using a consistent method (pain VAS). It is notable that this is not consistently undertaken in other trials, and consequently systematic reviews of DMARDs provide only incomplete evidence that they reduce pain with many studies not measuring pain [33]. Trials of treat-to-target strategies are ideally placed to demonstrate directly that targeting remission/LDA reduces pain intensity scores. Three treat-to-target trials comparing intensive treatment with standard care reported significant benefits of intensive management on pain [20, 34, 35]. However, other trials of treat-to-target strategies either compared different active treatment strategies which had similar impacts on pain or failed to report pain [36]. While these trials alone do not provide definitive evidence that treat-to-target optimises pain, when considered with our current secondary analysis and other observational studies in this area, the evidence in favour of targeting remission/LDA to control pain becomes highly persuasive.

We used a 100-mm VAS to measure pain intensity. This quantitative outcome measure is widely used to measure pain in RA trials and represents the primary outcome measure in many trials of chronic pain treatments [37]; it is also reliable and valid [38]. Its chief limitation is that it only captures one aspect (intensity) of a multidimensional problem (pain). Numerous other quantitative instruments exist that capture the broader aspects of RA pain [39], such as the RA Pain Scale, which contains 24 items measuring the physiological, affective, sensory-discriminative, and cognitive aspects of pain [40]. However, these more comprehensive tools are not widely used in RA trials, with a previous systematic review of RA trial patient-reported outcome measure use reporting that amongst 250 studies only 40% reported pain, of which 89% used the pain VAS or numeric rating scale [41]. Furthermore, the subjective nature of a person’s pain experience (which cannot be directly observed by clinicians and researchers) has led Wideman et al. to propose the use of a multimodal assessment model of pain [42], which incorporates both qualitative methods to evaluate the pain experience and pain expression in addition to more widely used quantitative methods. This model may change how pain in RA is assessed and managed in the future, with further research required to determine the extent to which controlling disease activity affects the broader aspects of pain (including its experience and expression) in patients with RA.

Our secondary analysis has several strengths. First, it assessed a large number of patients with RA from multiple English centres (optimising generalisability). Second, its key findings were replicated across patient groups (early and established RA, active and inactive disease) increasing their reliability. Third, the trials used rigorous highly standardised approaches to data collection. It also has important limitations. First and foremost, the secondary analyses were not pre-specified at study conception; consequently, we have not tested specific pre-defined hypotheses. Second, disease activity was evaluated using the DAS28-ESR. The findings may have differed if other composite assessments were used such as the simple disease activity index and the clinical disease activity index, which also include assessors’ global assessments [43]. Four of the trials and studies (CARDERA1, TITRATE, OPTTIRA and REMIRA) recorded 12-month assessors’ global assessments; Spearman’s correlations showed these had weaker associations with pain intensity scores than PGA but stronger than joint counts. Third, only patients with complete data at 0, 6 and 12 months were analysed. Analysing different data points may give a different picture. In particular, as pain intensity can change quite rapidly over time, the overall temporal relationships may be more complex. Fourthly, only a single pain dimension, pain intensity, was assessed. Further research is needed to establish why some patients with active RA have little pain while some patients in remission have moderately high pain intensities. Co-existing depression [44] or fibromyalgic rheumatoid may contribute [45, 46], though recent research suggests pain mechanisms are different in RA and fibromyalgia [47]. Finally, we did not consider differences between the various types of drug used. Conventional DMARDs, biologic DMARDs and glucocorticoids may all have different effects on pain intensity but our analyses has not evaluated this possibility.

## Conclusions

Our results strongly support the EULAR inflammatory arthritis pain management guideline recommendation that the initial crucial step in managing RA pain is controlling disease activity [5]. They also indicate that pain itself is an integral part of “disease activity” with PtGA acting as a reasonable proxy for pain intensity in many, but not all, patients with RA. Given the substantial impact of disease activity on pain intensity, the benefits of other management approaches to control pain need to be evaluated in a manner that takes into account disease activity. Finally, the impact of reducing disease activity on pain intensity levels, particularly in patients with high initial pain intensities, appears far larger than that which can be achieved using non-steroidal anti-inflammatory drugs or analgesics, including opioids [48]. It therefore seems crucial to focus on reducing disease activity with disease-modifying drugs to control pain before considering additional drug therapies.

## Data Availability

The datasets analysed during the current study are not publicly available but may be available from the corresponding author on reasonable request.

## Abbreviations

BMI: Body mass index
CARDERA-1: Combination Anti-rheumatic Drugs In Early Rheumatoid Arthritis 1 Trial
CARDERA-2: Combination Anti-rheumatic Drugs In Early Rheumatoid Arthritis 2 Trial
CI: Confidence interval
DAS28-ESR: Disease Activity Score for 28 joints using ESR
ESR: Erythrocyte sedimentation rate
HDA: High disease activity
LDA: Low disease activity
MDA: Moderate disease activity
OPTTIRA: Optimising Treatment with Tumour Necrosis Factor Inhibitors in Rheumatoid Arthritis Trial
PtGA: Patient Global Assessment
RA: Rheumatoid arthritis
REMIRA: Remission In Rheumatoid Arthritis Study
SJC: Swollen joint count
TACIT: Tumour Necrosis Factor Inhibitors Versus Combination Intensive Therapy Trial
TITRATE: Treatment Intensities and Targets in Rheumatoid Arthritis Therapy Trial
TJC: Tender joint count
VAS: Visual analogue score
